# Epidemiological profile and antifungal susceptibility pattern of *Trichosporon* species in a tertiary care hospital in Chandigarh, India

**DOI:** 10.18502/cmm.7.1.6179

**Published:** 2021-03

**Authors:** Vibha Mehta, Jagdish Chander, Neelam Gulati, Nidhi Singla, Hena Vasdeva, Raman Sardana, Awadhesh Kumar Pandey

**Affiliations:** 1 Department of Microbiology, Government Medical College Hospital, Chandigarh, India; 2 Department of Microbiology, Indraprastha Apollo Hospitals, New Delhi, India; 3 Department of Radiotherapy, Government Medical College Hospital, Chandigarh, India

**Keywords:** Antifungal susceptibility, Invasive trichosporonosis, Minimum inhibitory concentration, *Trichosporon*, Voriconazole

## Abstract

**Background and Purpose:**

*Trichosporon* species are ubiquitous in nature which are associated with fatal opportunistic invasive infections, especially in immunocompromised patients. The present study aimed to evaluate the epidemiological and clinical details, as well as the antifungal susceptibility pattern of the patients with *Trichosporon* infections.

**Materials and Methods:**

In total, 50 clinical isolates of *Trichosporon species* from various samples were included in this study. The samples were isolated over a period of 18 months from patients in a tertiary hospital in North India. The isolates were characterised phenotypically with Vitek MS (bioMérieux, France). *Trichosporon* spp. were isolated from urine (30%), nail (30%), tissue (16%), pleural fluid (14%), and sputum (5%). In total, majority of the isolates were of *Trichosporon asahii* (92%), followed by *Trichosporon mucoides* (6%), and *Trichosporon ovoides* (2%). It is noteworthy that most of the reported cases were from intensive care unit (34%).

**Results:**

Intravenous catheters, antibiotics, and antifungal uptake were significantly associated risk factors with *Trichosporon* infection. All invasive isolates were observed to be resistant *in vitro* to caspofungin and exhibited high minimum inhibitory concentration (MIC) values against amphotericin B, fluconazole, and 5-flucytosine. The MICs for voriconazole and posaconazole were low.

**Conclusion:**

Trichosporonosis is being increasingly reported all around the world, including India. The results of this study highlighted the importance of early detection and treatment for this emerging yeast and also added to the ongoing surveillance for the antifungal susuceptibility pattern for this fungus.

## Introduction

*Trichosporon* species was first discovered in 1865 as a benign infection from a patient with white Piedra. Currently, *Trichosporon* species have been increasingly recognized as opportunistic pathogens capable of causing invasive diseases, especially in immunosuppressed patients [ 1 ]. Multiple species of the genus *Trichosporon* were used to be called collectively *T. beigelii*, while currently, the genus *Trichosporon* comprises 50 species with 16 human pathogens [ 2 , 3 ].

In the past three decades, *Trichosporon* species have been implicated in invasive infections in the immunocompromised hosts. Moreover, there have been rare reports of trichosporonosis in immunocompetent patients [ 4 ]. Most cases have been reported among neutropenic patients with hematological or solid organ malignancies, and bone marrow or solid organ transplantation. Other patients at risk for invasive disease include patients with AIDS, extensive burns, intravascular catheters; patients who receive corticosteroids or undergo heart valve surgery and liver transplantation; and patients on dialysis [ 4 ].

*Trichosporon* species exhibit variable MICs against amphotericin B and moderate susceptibility to fluconazole and itraconazole [ 5 ]. This fungus exhibits intrinsic resistance to echinocandins, as evidenced by the high MICs reported until now, and the reports of breakthrough infections among patients receiving these antifungals [ 6 ]. The clinical picture of trichosporonosis resembles that of invasive candidiasis, a neutropenic patient with an acute febrile illness not responding to empirical broad-spectrum antibiotics or even empirical antifungal agents. The patient may rapidly develop multiorgan failure and become septic. According to the literature review, the prognosis is dismal as the case fatality rate is as high as 77% [ 7 ].

Lack of background knowledge impairs the proper diagnosis and treatment of *Trichosporon* species. The majority of the studies on this disease in India and other countries around the world are either retrospective or case reports. Therefore, there is a need for prospective studies, especially in developing countries, like India, due to the increase in the number of cases as well as the presence of diverse risk factors in both immunocompetent and immunocompromised patients [ 4 ]. Such studies can facilitate the timely diagnosis, analysis, and documentation of various risk factors and improve the treatment of the patient based on the reported MICs of the isolates. The present study aimed to investigate the epidemiology of this disease, including the clinical presentation with associated risk factors and antifungal susceptibility.

## Materials and Methods

This prospective observational study was conducted on all the samples received in the Mycology Laboratory wherein *Trichosporon* species had been isolated for 18 months (from January 2016 to June 2017). The samples included blood, urine, pus, sputum, peritoneal fluid, hair, nail, skin scrapings, or any other body fluid collected from both admitted and outpatients that were suspected of any fungal infection. A detailed pro forma was filled with various information about the demographic characteristics of the patients, fungal isolates, antifungal susceptibilities, and the final outcome of the patients. On the basis of sample site and presentation, we broadly categorized our cases into invasive and superficial trichosporonosis, similar to Colombo et al. [ 4 ].

In total, 50 patients of all age ranges from whom Trichosporon was isolated on culture were included in the study. Slide and tube KOH mounts were prepared with 10-20% KOH and examined for the presence of fungal elements, such as arthroconidia, blastoconidia, hyphae, or pseudohyphae [ 8 ]. Samples were inoculated for culture on Sabouraud dextrose agar (SDA) (HiMedia, India) tube slants with chloramphenicol and gentamicin both with and without cycloheximide . Each medium was inoculated in duplicate and incubated at 25°C and 37°C. The subcultures from blood culture bottles were performed on respective SDAs [ 8 , 9 ].

The SDA slants were observed for fungal growth daily for one week and twice a week for the next three weeks. The fungal growth was identified and assessed conventionally by standard mycological methods on the basis of macroscopic morphological features, a microscopic examination by lactophenol cotton blue mount, cornmeal morphology, and urease and sugar assimilation tests [ 10 ]. Confirmatory detection was performed with Vitek MS (bioMérieux, France), a commercially available MALDI-TOF MS platform [ 11 ]. 

Antifungal susceptibility testing was performed by micro-broth dilution technique for amphotericin B, 5-flucytosine, fluconazole, itraconazole, voriconazole, posaconazole, and caspofungin according to the Referencence Method for Broth Dilution Antifungal Susceptibility Testing of Yeast by the Clinical Laboratory Standard Institute (CLSI) (M27-A3). [ 12 ]. Moreover, the *C. parapsilosis* ATCC 22019 and *C. krusei* ATCC 6258 were used as quality strains. The tested antifungals were provided in the form of powders by a commercial source (Sigma-Aldrich, USA). The MICs were read after 24 h of incubation at 37°C.

The association between categorical variables was determined using Fisher's exact test. A two-sided p-value of less than 0.05 was considered statistically significant. The study was conducted after obtaining ethical approval from the Ethical Clearance Committee of the Government Medical College and Hospital, Chandigarh, India. The present research was conducted based on the ethical guidelines for biomedical research on human subjects According to the Central Ethics Committee on Human Research of Indian Council of Medical Research, New Delhi, India in 2006 [ 13 ] and the Declaration of Helsinki of 2008 [ 14 ].

## Results

During the study period of 18 months, 2405 samples were received in the mycology laboratory. In total, 675 samples were positive for various fungal isolates with 50 (7.4%) samples yielding the growth of *Trichosporon* species. 15 (30%) and 35 (70%) out of the 50 *Trichosporon* isolates belonged to the superficial and invasive Trichosporonoses. Most of the isolates were isolated from urine (n=15, 30%) and nail (n=15, 30%), followed by tissue (n=8, 16%). Besides, 14% (n=7) and 5% (n=5) of cases were reported from pleural fluid and sputum, respectively. 

**Figure 1:**
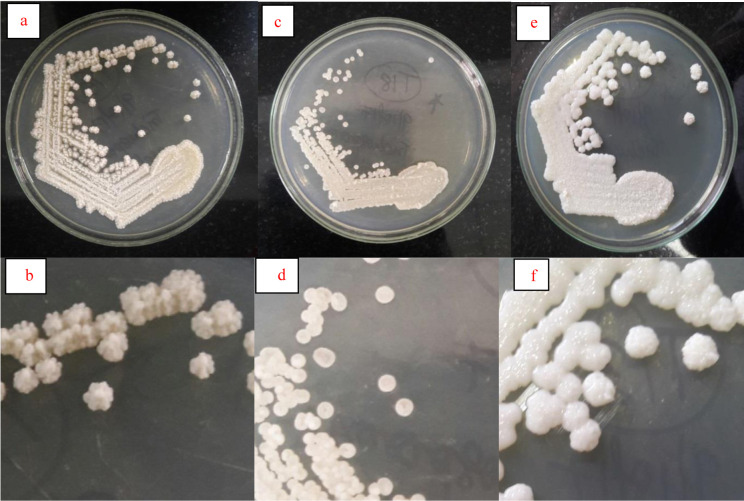
Colony morphology on Sabouraud dextrose agar (SDA) after 48 h of incubation at 37ºC of *Trichosporon asahii* (a, b): Dry and highly wrinkled colony morphology of *Trichosporon ovoides* on SDA (c, d): Mucoid and shiny colony morphology of *Trichosporon mucoides* on SDA (e, f):

*Trichospron asahii* was the most commonly isolated species (46, 92%) while only 6% (n=3) and 2% (n=1) of the isolates were *Trichosporon mucoides* and *Trichosporon ovoides*. All the cases of invasive trichosporonosis were caused by *T. asahii* (100%) and it was also the commonest species in superficial trichosporonosis (11 out of 15 cases, 73%). The three *T. mucoides* (3 out of 15 cases, 20%) and one *T.ovoides* (1 out of 15 cases, 7%) were isolated from cases of onychomycosis, belonging to superficial trichosporonosis (Figure 1 shows the colony morphologies of the three species of *Trichosporon*).

The mean and median age of the patients were 49.5±11.3 and 45 years, respectively, Furthermore, the minimum and maximum ages of the cases were 21 and 70 years, respectively. The maximum number of patients was within the age range of 41-50 (17/50, 34%) for both invasive (12 out of 35 cases, 34.2%) and superficial trichosporonosis (5 out of 15 cases, 33.33%). Proportion of male patients was equal to that of female patients (25 out of 25 cases, 1:1). Invasive trichosporonosis was more common in males (21 out of 35 cases, 60%), while superficial trichosporonosis was more observed in females (11 out of 15 cases, 73%).

Maximum reported cases were from intensive care unit (ICU) (n=17, 34%), followed by dermatology OPD (n=15, 30%), Medicine Ward (n=10, 20%), and Surgery Ward (n=8, 16%). Amongst the various studied risk factors, antibiotics intake (35, 70%), antifungal intake (n=9, 18%), and intravenous catheters (n=34, 68%) were significantly associated with *Trichosporon* infection. Invasive trichosporonosis had a significant association with the history of antibiotics intake (35 out of 35 cases, 100%), usage of urinary catheter (22 out of 35 cases, 62.8%), ICU stay (17 out of 35 cases, 48.5%), underlying malignancy (6 out of 35 cases, 17.1%), bronchial asthma with chronic obstructive pulmonary disease (COPD) (6 out of 35 cases, 17.1%), immunosuppressant use (5 out of 35 cases, 14.2%), and surgery in the last three months (3 out of 35 cases, 8.5%). Superficial trichosporonosis was significantly associated with a history of antifungal intake (itraconazole) (4 out of 15 cases, 26.6%) (Table 1).

**Table1:** Risk factors associated with Trichosporonosis

Risk factor	Trichosporonosis		Invasive Trichosporonosis		Superficial Trichosporonosis	
	Number	*p-value*	Number	*p-value*	Number	*p- value*
Neutropenia	7	>0.05	7	>0.05	0	>0.05
Malignancy	6	>0.05	6	<0.05	0	>0.05
Intra- venous catheters	34	<0.05	34	>0.05	0	>0.05
Urinary catheters	22	>0.05	22	<0.05	0	>0.05
Central venous catheters	6	>0.05	6	>0.05	0	>0.05
Immunosuppressant intake	5	>0.05	5	<0.05	0	>0.05
Steroid intake	13	>0.05	13	>0.05	0	>0.05
Surgery	3	>0.05	3	<0.05	0	>0.05
Dialysis	5	>0.05	5	>0.05	0	>0.05
Intensive care unit stay	17	>0.05	17	<0.05	0	>0.05
Antifungal intake	9	<0.05	5	>0.05	4	<0.05
Antibiotics intake	35	<0.05	35	<0.05	0	>0.05
History of diabetes mellitus	17	>0.05	16	>0.05	1	>0.05
Bronchial asthma with chronic obstructive pulmonary disease	6	>0.05	6	<0.05	0	>0.05
History of tuberculosis	2	>0.05	2	>0.05	0	>0.05

Table 2 summarizes the MIC range, MIC_50_, and MIC_90_ of Trichosporon species against various antifungals tested in this study. Voriconazole and posaconazole had lower MICs and all isolates were

**Table2:** Minimum inhibitory concentration of various *Trichosporon* isolates

Antifungal agent		*Trichosporon*	*T. asahii*	*T. asahii* (Invasive)	*T. asahii (Superficial)*	*T. mucoides*	*T. ovoides*
Amphotericin-B (µg/ml)	MIC range	0.25-≥16	0.25-≥16	0.25-≥16	0.5-≥16	8-≥16	≥16
	MIC_50_	16	16	16	16		
	MIC_90_	16	16	16	16		
	G mean	6.964	6.964	6.964	4.832	13.125	
5-Flucytosine (µg/ml)	MIC range	0.125-≥64	0.125-≥64	0.125-≥64	0.125-≥64	0.125-2	≥64
	MIC_50_	2	2	4	2		
	MIC_90_	8	8	16	4		
	G mean	2.234	2.234	2.234	2.858	1.811	
Fluconazole (µg/ml)	MIC Range	≤0.125-32	≤0.125-32	≤0.125-32	≤0.125-4	≤0.125-4	≤0.125
	MIC_50_	4	4	4	4		
	MIC_90_	8	8	32	4		
	G mean	2.567	2.567	2.567	3.108	1.486	
Itraconazole (µg/ml)	MIC range	≤0.0313-0.25	0.125-0.25	≤0.0313-0.25	≤0.0313-≤0.0313	≤0.0313-≤0.0313	≤0.0313
	MIC_50_	0.0313	0.0313	0.0313	0.0313		
	MIC_90_	0.0625	0.125	0.125	0.0313		
	G mean	0.041	0.041	0.041	0.046	0.0313	
Voriconazole (µg/ml)	MIC range	≤0.0313-0.5	≤0.0313-0.5	≤0.0313-0.5	≤0.0313-0.0625	≤0.0313-≤0.0313	≤0.0313
	MIC_50_	0.0313	0.0313	0.0313	0.0313		
	MIC_90_	0.0625	0.25	0.125	0.0313		
	g mean	0.038	0.038	0.038	0.043	0.0313	
Posaconazole (µg/ml)	MIC range	≤0.0313-2	≤0.0313-2	≤0.0313-2	≤0.0313-0.5	≤0.0313-≤0.0313	≤0.0313
	MIC_50_	0.125	0.125	0.125	0.125		
	MIC_90_	0.5	0.5	1	0.25		
	G mean	0.128	0.128	0.128	0.164	0.093	
Caspofungin (µg/ml)	MIC Range	≤0.125-16	≤0.125-16	≤0.125-16	≤0.125-8	≤0.0125-4	≤0.125
	MIC_50_	8	8	8	4		
	MIC_90_	8	8	8	8		
	G mean	3.531	3.531	3.531	4.443	2.438	

MIC: Minimum inhibitory concentration, G mean: geometric mean

resistant to all caspofungin. Most strains exhibited relatively high MIC values against amphotericin B, fluconazole, and 5- flucytosine. 

## Discussion

Trichosporonosis is an emerging cause of morbidity and mortality in both developing as well as developed countries [ 4 ]. *Trichosporon* species have been increasingly recognized as opportunistic pathogens capable of causing invasive disease, especially in immunosuppressed patients [ 8 ]. Population of immunocompromised patients is on the rise which means that the fraction of at-risk population is increasing making more people prone to the development of this disease [ 6 ]. This is consistent with an increasing incidence rate of disseminated *Trichosporon* infections in humans alongside other invasive fungal infections [ 8 ].

On the basis of presentation and sample site, we broadly categorized our cases into invasive and superficial trichosporonosis, similar to Colombo et al. [ 4 ]. Based on the results, 35 (70%) out of the 50 *Trichosporon* isolates included in this study were of invasive trichosporonosis, most of which were isolated from urine (n=15, 30%). Furthermore, 30% (n=15) of the isolates were superficial trichosporonosis, all of which were isolated from the nail. In 2015, Montoyo et al. also observed a similar sample distribution, wherein 26 (66%) out of the total 39 cases were from urine and only five of them (12%) were from nail samples [ 15 ]. It is noteworthy that urine was the commonest sample in other studies as well [ 16 , 17 ]. India has a hot humid climate which could be accounted for the high number of onychomycosis cases in this study since it facilitates the growth of this fungi.

Although the male to female ratio of the total subjects was 1:1, most of the cases of invasive trichosporonosis were male (60%). Nevertheless, this ratio was opposite regarding superficial trichosporonosis since females accounted for 73% of cases. The male preponderance in invasive trichosporonosis was reported by most studies, while Colombo et al. in their study reported female preponderance in superficial trichosporonosis [ 4 , 18 , 19 ]. In the present study, the mean age of the participants was 49.5±11.32 years. Maximum number of cases (n=17, 34%) were observed in the age range of 41-50 years both in invasive and superficial trichosporonosis. Yang et al. and Wei sun et al. reported the maximum number of invasive and superficial trichosporonosis cases in the age ranges of ≥ 66 years and 70 years, respectively [ 16 , 19 ]. Besides, in the present study, trichosporonosis was mostly found in the younger age group which clearly indicates the growing severity of the disease.

Maximum reported cases (34%) in this study and some previous studies were from ICU [ 18 , 20 ]. Based on the findings, trichosporonosis had a significant relationship with antibiotic and antifungal intake and intravenous catheters (P≤0.05). Moreover, it was found that invasive trichosporonosis had a significant association with malignancy, surgery, bronchial asthma with COPD, urinary catheter, antibiotic and immunosuppressant intake, and ICU stay. In addition, antifungal intake (itraconazole) was significantly associated with superficial trichosporonosis.

Based on risk factor analysis, the history of antibiotic intake was observed in 100% and 82.2 % of patients in other studies [ 18 , 19 ]. In the present study, 70% of subjects had a history of antibiotic intake. Moreover, corticosteroid intake history was observed in 26% of cases in this research and 34.1% and 53% of patients in other studies [ 18 , 20 ]. In addition, immunosuppression history was observed in 31.7% of participants in a study performed by Almeida junior et al. while in this study 10% of subjects had a history of immunosuppression [ 18 ]. In a study carried out by Wei sun et al., 60.8% of cases used a urinary catheter, while in the present study 44% of patients had catheterization [ 19 ]. Furthermore, in the aforementioned study, a history of diabetes was seen in 34.7% of cases which was the same in the present study [ 19 ].

It can be said that *T. asahii*, *T. asteroides*, *T. cutaneum*, *T. inkin*, *T. mucoides*, and *T. ovoides* are common human pathogens causing superficial and disseminated infections [ 21 ]. More specifically, *T. asahii* and *T. mucoides* appear to be much more common in cases of systemic mycosis in immunocompromised patients. Moreover, *T. inkin* and *T. ovoides* are associated with pubic white piedra and white piedra of the head, respectively, while *T. asteroids* and *T. cutaneum* are associated with superficial skin lesions [ 4 , 21 ].

It must be mentioned that *T. asahii* was the only species isolated from all the cases of invasive trichosporonosis in this study. Moreover, it was the most common species in superficial trichosporonosis, accounting for 92% of cases. Other isolated species were *T. mucoides* (n=3, 6%) and *T. ovoides* (n=1, 2%). Similar results were found by Almeida et al. who also reported *T. asahii* as the most common species in urine samples of hospitalized patients [ 18 ]. Taverna et al. carries out a study on 41 subjects and found *T. asahii* in 70% of them [ 22 ]. 

Arabatzis et al. performed a study in Athens and had a similar observation, reporting *T. asahii* as the leading cause of infection with 88% prevalence [ 23 ]. Yang et al. found 68.1% cases of *T. asahii* in their study [ 16 ]. Furthermore, Kalkanci et al. in their study found that 81.3% of cases were infected by *T. asahii* [ 17 ]. Based on previous studies, there is consistency in clinical dominance of *T. asahii* in Brazil, China, Japan, Spain, Taiwan, Thailand, and Turkey118 [ 16 ]. 

Rastogi et al. found *T. debeurmannianum*, a rare species of Trichosporon, isolated from nail. Moreover, they reported three (9.6%) and one (3.2%) cases of *T. ovoides* and *T. mucoides*, respectively, none of which were from nail [ 24 ]. Singh et al. reported one (4.1%) *T. ovoides* isolated from a case of superficial trichosporonosis and one (4.1%) *T. mucoides* isolated from invasive trichosporonosis [ 25 ].

Until now, there have been no recommendations on MIC breakpoints for *Trichosporon* from the two main consortia, CLSI and EUCAST [ 9 ]. The MICs for *T. asahii* have been discussed here as it was the main isolated species in this study. Distribution of MICs against amphotericin B is quite heterogeneous. Higher MICs have been described by various studies carried out by Rastogi et al. (0.25-≥64 µg/ml) [ 24 ], Montoya et al. (0.5-16 µg/ml) [ 15 ]. Besides, even the present study produced similar results with MIC values of 0.125-16 µg/ml. The MIC_50_ and MIC_90_ had wide ranges in various studies such as 0.5-16 µg/ml and 2-16 µg/ml, respectively [ 16 , 24 ]. 

The strains in this study had very high MIC_50_ (16 µg/ml) and MIC_90_ (16µg/ml) values. The MIC values for *T. mucoides* (8-≥16 µg/ml) and *T. ovoides* (≥16 µg/ml) were also high. It must be noted that 5-flucytosine is not usually a preferred medication for Trichosporon. The MIC values of 5-flucytosine were high in this study (0.125-≥16 µg/ml), similar to those in the study performed by Kalkanci et al. (0.125-32 µg/ml) and [ 17 ] Lemes et al. (0.25-32 µg/ml) [ 5 ]. Montoya et al. reported quite higher MICs of 4-64 µg/ml [ 15 ].

Among the azoles, fluconazole is the most commonly used medication. The MIC distribution of fluconazole is also heterogeneous in various studies, with wide ranges of MIC, MIC_50_, and MIC_90_. The MIC of fluconazole in this study was ≤ 0.125-32 µg/ml which was higher in most other studies, such as the ones conducted by Kalkanci et al. (4-64 µg/ml) [ 17 ], Rastogi et al. (2-≥64 µg/ml) [ 24 ], Arabatzis et al. (1-64 µg/ml) [ 23 ], and Taverna et al. (1-64 µg/ml) [ 22 ].

The MIC_50_ of fluconazole in this research was 4 µg/ml while in other studies it was within the range of 0.5-8 µg/ml [ 5 , 19 ]. The MIC_90_ in this study was 8 µg/ml which has been reported to be as high as 64 µg/ml in one of the previous studies [ 23 ]. The *T. mucoides* (0.125-4 µg/ml) and *T. ovoides* had lower MICs (≤0.125 µg/ml) in this study. Iitraconazole had lower MICs (0.125-0.25 µg/ml) which has also been observed by others [ 17 , 19 , 22 ], while few studies have reported it to have higher MICs [ 23-25 ].

Lower MICs were also observed for triazoles, voriconale, and posaconale. The MIC for voriconazole was 0.0313-0.5 in the present study, and other researchers have also reported lower MICs [ 15-17 , 19 , 22 , 25 ]. However, Arabatzis et al. (0.64-32 µg/ml) [ 23 ] and Rastogi et al. (0.12-4 µg/ml) [ 24 ] found higher MICs for voriconazole. Moreover, they reported the MIC_90_ of 32 and 4 µg/ml for voriconazole, respectively, which are much higher than the obtained values in this study. The same authors also reported higher MICs for posaconazole, 0.032-16 µg/ml [ 23 ] and 0.25-4 µg/ml [ 24 ], respectively, which are again higher than those in this study (0.031-2 µg/ml). 

It must be noted that the results of other studies in this regard are similar to those of the present research [ 15 , 22 ]. *Trichosporon* has been considered inherently resistant to caspofungin with constantly elevated MICs [ 4 , 18 ]. It showed higher MICs in this research similar to other studies [ 15 , 18 ].

## Conclusion

Results of the present study emphasize the dynamic nature of *Trichosporon* spp. in the immunocom-promised and immunocompetent hosts. Their frequency raises the concern of the increase of *Trichosporon*. This study will add to the ongoing surveillance of antifungal susceptibility patterns. Interval surveillance of this type is an essential component in the development of institutional guidelines for prophylaxis, empiric, or pre-emptive therapy for such life-threatening infections. 

## Authors’ contribution

J.C. and N.G. were involved in study conception and design. V.M., N.G., A.K.P, H.V. and R.S. conducted the study. V.M., N.G., N.S. were involved in data compilation, analysis, and manuscript preparation. J.C., A.K.P, and N.S. did the proofreading and corrections of the manuscript. The manuscript has been read and approved by all the authors. 

The manuscript has been read and approved by all the authors.

## Financial disclosure

This research received no external funding.
